# Kaolin Film Increases Gas Exchange Parameters of Coffee Seedlings During Transference From Nursery to Full Sunlight

**DOI:** 10.3389/fpls.2021.784482

**Published:** 2022-01-07

**Authors:** Deivisson Pelegrino de Abreu, Newton de Matos Roda, Gideao Pelegrino de Abreu, Wallace de Paula Bernado, Weverton Pereira Rodrigues, Eliemar Campostrini, Miroslava Rakocevic

**Affiliations:** ^1^Laboratory for Plant Genetic Breeding (LMGV), State University of the North Fluminense Darcy Ribeiro, Rio de Janeiro, Brazil; ^2^Department of Exact, Environmental and Technological Sciences (CEATEC), Pontifical Catholic University of Campinas, Campinas, Brazil; ^3^Business School and Polytechnic School, MBA in Business Technology, Data Science and Big Data, Pontifical Catholic University of Rio Grande do Sul, Porto Alegre, Brazil; ^4^Center of Agricultural, Natural and Literary Sciences, State University of the Tocantina Region of Maranhão (UEMASUL), Estreito, Maranhão, Brazil

**Keywords:** chlorophyll fluorescence, leaf photosynthesis, leaf transpiration, thermography, water management

## Abstract

Increases in water use efficiency (WUE) and the reduction of negative impacts of high temperatures associated with high solar radiation are being achieved with the application of fine particle film of calcined and purified kaolin (KF) on the leaves and fruits of various plant species. KF was applied on young *Coffea arabica* and *Coffea canephora* plants before their transition from nursery to full sunlight during autumn and summer. The effects of KF were evaluated through the responses of leaf temperature (T_leaf_), net CO_2_ assimilation rate (*A*), stomatal conductance (*g*_s_), transpiration (*E*), WUE, crop water stress index (CWSI), index of relative stomatal conductance (I_g_), initial fluorescence (F_0_), and photosynthetic index (PI) in the first 2–3 weeks after the plant transitions to the full sun. All measurements were performed at midday. In *Coffea* plants, KF decreased the T_leaf_ up to 6.7°C/5.6°C and reduced the CWSI. The plants that were not protected with KF showed lower *A*, *g*_s_, *E*, and I_g_ than those protected with KF. *C. canephora* plants protected with KF achieved higher WUE compared with those not protected by 11.23% in autumn and 95.58% in summer. In both *Coffea* sp., KF application reduced F_0_, indicating reduced physical dissociation of the PSII reaction centers from the light-harvesting system, which was supported with increased PI. The use of KF can be recommended as a management strategy in the transition of *Coffea* seedlings from the nursery shade to the full sunlight, to protect leaves against the excessive solar radiation and high temperatures, especially in *C. canephora* during the summer.

## Introduction

The fine particle film of calcined and purified kaolin (KF) application is a technological product-oriented to sustainable use of water resources in various agricultural crops (Boari et al., 2014; Brito et al., 2018; Faghih et al., 2019). The use of KF is certified by the Organic Materials Review Institute (OMRI, 2021) as an authorized substance in organic food production, in the context of rising health and environmental concerns, especially for organically growing agricultural and horticultural crops (Sharma et al., 2015; Mphande et al., 2020). Adding to improved crop water management, KF can be applied to reduce the impact of heat and excessive light (Steiman et al., 2007; Santos et al., 2021) and also help in pest (Amalin et al., 2015) and pathogen control (Glenn et al., 2001; Tubajika et al., 2007). Fine kaolin films reduce negative UV impacts by increasing light reflection in apple, *Malus domestica* (Glenn et al., 2002); reduce leaf temperature by 1.1°C in tomato, *Solanum lycopersicum* (Boari et al., 2014); reduce water stress in pepper, *Capsicum annuum* (Creamer et al., 2005); increase water use efficiency (WUE) by 26% in tomato (AbdAllah, 2019), and stomatal conductance (*g*_s_) in well-watered grapevines, *Vitis vinifera* (Glenn et al., 2010); increase leaf photosynthesis (*A*) in apple trees (Glenn et al., 2001); increase the sucrose biosynthesis in grapevines (Conde et al., 2018); increase height and diameter growth in young eucalyptus hybrid plants, *Eucalyptus grandis* × *Eucalyptus urophylla* (Santos et al., 2021). In tomato cultivation, the KF application results in a 23% increase of a marketable yield (average of 3 years) with significant net profit gain for the tomato producer of 600 € ha^–1^ on non-saline soils, and 900 € ha^–1^ in saline soils (Boari et al., 2014).

Coffee is one of the most consumed world beverages, and its production is made possible by the work of approximately 100 million coffee growers worldwide (Davis et al., 2019). Among 124 species of wild coffees, the global coffee trade relies on two species, namely Arabica (*Coffea arabica*) and Robusta coffee (*Coffea canephora*). The world’s two largest coffee producers, Brazil, and Vietnam are geographically distant from the African centers of coffee origin (Anthony et al., 2011). The environmental pressure of monoculture, the predominant Brazilian coffee-growing system (DaMatta et al., 2019) is far from deep forest environmental conditions in the centers of coffee origin, which causes their classification into shade-tolerant species (Ayalew, 2018). The *C. arabica* is an understory tree and an endemic species originated at the Ethiopian rainforests at altitudes above 1,500 m characterized with an average annual temperature of 20°C; *C. canephora* originated at West and Central Africa of altitudes from 0 to 1,200 m, characterized with average annual temperature between 24 and 26°C, where it evolved as a medium-sized tree (Charrier and Berthaud, 1985).

The expectation of the life of a coffee tree in their natural habitat is up to 100 years, while in plantations it is about 30–40 years (Gokavi et al., 2019). In plantations, tree production and yield cycles are regulated by diverse systems of training and renovations (Taugourdeau et al., 2014; Rakocevic et al., 2021a). In Brazil, about 300,000 ha are planted every year for orchard renovation or for the new area’s plantation, which corresponds to 13% of the areas under coffee crops (CONAB, 2021).

The coffee seedlings are produced in nurseries, normally covered with plastic mesh, which can block about 50 to 75% of photosynthetically active radiation (PAR), attaining up to 600–700 μmol m^–2^ s^–1^ at midday of one tropical sunny day (Matiello et al., 2010). Under actual air CO_2_ concentration, this PAR range is considered as a light saturation point for *C. arabica* (Rodrigues et al., 2016; Rakocevic et al., 2021b). High light intensities provoke photoinhibition in both *C. arabica* and *C. canephora*, decreasing maximum photochemical efficiency (F_v_/F_m_) because of an increased initial and a quenched maximum fluorescence (DaMatta and Maestri, 1997; Martins et al., 2014). In coffee, generally limited and low *A* is explained by stomatal factor limitations, followed by the mesophyll and biochemical constraints (Martins et al., 2014). Moreover, coffee plants showed significant sensibility to UV radiation, mainly *C. canephora*, which displayed reduced root and total biomass, number of leaves and leaf area, increased leaf elongation rate under ambient compared with reduced UV radiation (Bernado et al., 2021).

The optimal development of *Coffea* seedlings in the nursery is dependent on water supply, with an average of 4.5 mm day^–1^, summing about 600 mm (Vallone et al., 2010). When the seedlings formed in the nursery reach 4 to 6 pairs of leaves, they are subjected to a gradual increase of light and eventually gradual water reduction for about 30 days in a process of acclimatization. Afterward, seedlings are planted to the field, which is recommended to occur in the rainy spring in not irrigated fields (Mesquita et al., 2016). Until they are 1 year old, the young plants in the field need to be irrigated (Gervásio and Lima, 1998), otherwise without the irrigation, the mortality of transplanted coffee seedlings attains up to 73% after 6–8 months (Oliveira et al., 2015).

In the post-planting period, under the combination of elevated air temperatures and elevated solar irradiance, especially its UV bands (Ulm and Jenkins, 2015; Bernado et al., 2021), one physiological disorder called leaf sunburn can occur in coffee seedlings (Santos et al., 2016). Sunburn is expressed by symptoms of chlorosis and necrosis in a great number of species (Racskó et al., 2010). Among deleterious effects of sunburn, the reduction of leaf gas exchange, reduction in plant height, leaf area, shortened internode, and branch length, and sunburn browning of fruits were reported in apple (Racskó et al., 2010) and coffee trees (Santos et al., 2016). Recently, KF application is shown to be the best method to prevent sunburn in fruits of pomegranate, *Punica granatum* (Yazici and Kaynak, 2006; Sharma et al., 2018).

Despite the great importance of the coffee crops in Brazil, and the continuous necessity of new sustainable strategies for agricultural production, natural resource conservation, and to guarantee the maximum setting of seedlings in the transition from the nursery to the field, no research was performed to test the use of KF on this crucial stage of the seedling’s establishment in the full sunlight. It was hypothesized that KF leaf protection from high light intensities and temperature stress can avoid the photoinhibition, improving leaf gas exchanges and WUE in young coffee plants, having more pronounced impacts on *C. canephora* than in *C. arabica*. Thus, the aim of this work was to assess the effects of KF technology on chlorophyll *a* fluorescence and net photosynthesis rate of young coffee plants in two seasons, autumn and summer, in their transition from nursery to full sun under the well-watered conditions, contributing to the conservation of local water resources, environmental sustainability, and increasing plant abilities to cope with abiotic stresses.

## Materials and Methods

### Experimental Site, Species Description, and Fine Calcined Kaolin-Based Particle Films Application

The experiment was conducted at the State University of Northern Rio de Janeiro, in Campos dos Goytacazes (21° 44′ 47″ S and 41° 18′ 24″ W, at 14 m altitude), Southeastern Brazil, using important cropped genotypes in Brazil: *C. arabica* L. cv. Catuaí Vermelho IAC 44, and *C. canephora* Pierre ex. A. Froehner, cl. Al. Seedlings of *C. canephora* and *C. arabica* were cultivated in 800-mL plastic bags in a nursery for 5 months, covered with a plastic mesh, which were blocked with 50–70% of PAR (Braun et al., 2007). When seedlings reached five pairs of leaves, we simulated the commercial planting by transplanting young plants into 25 L pots under the shade of plastic mesh and then under full sunlight. At the bottom of each pot, 3 L of gravel was placed before adding the substrate to facilitate the water drainage. The substrate in both plastic bags and 25 L pots was composed of sieved oxisol, sand, and fermented cattle manure in a 7:1:2 ratio. Two kilograms of dolomitic limestone and 7 kg of simple superphosphate were added to 1,000 L of the substrate.

The KF used in the experiment was produced from calcinate purified kaolin (Surround^®^ WP; TK Inc., Phoenix, AZ., United States), enriched and with a low abrasive compound of aluminum silicate [Al_4_Si_4_O_10_(OH)_8_], which is chemically inert and highly soluble in water (Glenn et al., 2010). The Surround^®^ WP was mixed in a 2-L beaker, in the proportion of 50 g of product to 1 L of water, which resulted, when applied on the leaves, in a coverage of 646 mg m^–2^ of leaves, predominantly on their adaxial side. The foliar application was performed with one 1.2 L capacity precompression sprayer. The amount of KF applied per meter square of leaves was obtained from the arrangement of Petri dishes, of known mass and area, positioned at the same angle as the leaves of *Coffea* sp. in the moment of KF application. After application, the Petri dishes were collected and left in an oven at a temperature of 85°C for 24 h to complete a water evaporation, and after that Petri dishes were weighed.

The experiment was carried out in two seasons, autumn of 2018 and summer of 2019. For ecophysiological measurements performed in autumn of 2018, seedlings were firstly transplanted into 25-L pots on March 7th and remained under the shade of nursery for 78 days. KF application was performed on May 20th. The shade was removed (simulating transplanting to the full sunlight of the field) on May 22nd at 8 p.m., and afterward the plants were exposed to full sunlight for 22 days. For ecophysiological measurements in the summer of 2019, the seedlings were transplanted into 25-L pots on March 10th and remained under the shade of nursery for two more days. The KF application was performed on March 10th. The shade was removed on March 12th at 8 p.m., afterward the plants were exposed to full sunlight for 14 days.

### Microclimate Description

The microclimate and ecophysiological measurements were conducted from the day 0 (representing seedling responses under shade nursery with 50% of PAR) to the 1st, 2nd, 7th, and 22nd/14th (autumn/summer) days of exposure to full sunlight (DFS), respectively.

Micrometeorological conditions, such as PAR (μmol m^–2^ s^–1^), air temperature (T_*air*_, °C), relative humidity (RH,%), and air vapor pressure deficit (VPD, kPa) were monitored using a miniautomatic climatological station (Model 2475, WatchDog Spectrum Technologies, Aurora, Illinois, United States) installed between the plants during the experiment. The data were recorded every 30 min, and data are presented as an average of three readings (i.e., readings shown for 8 a.m. were calculated as an average of those registered at 7:30, 8:00, and 8:30 a.m., Table 1).

**TABLE 1:** Photosynthetic active radiation (PAR), relative humidity (RH), air temperature (T_*air*_), and vapor pressure deficit (VPD) recorded for days of ecophysiological measurements of young coffee plants transferred from nursery to full sunlight.

		PAR (μmol m^–2^ s^–1^)					RH (%)					T (°C)					VPD (kPa)				
	DFS	0	1	2	7	22	0	1	2	7	22	0	1	2	7	22	0	1	2	7	22
Autumn	8 a.m	54	243	594	531	517	97	99	79	96	91	17	19	22	21	22	0.06	0.02	0.56	0.10	0.24
	10 a.m	259	1,232	1,482	1,232	1,064	55	68	57	69	70	25	24	27	25	25	1.43	0.95	1.53	0.98	0.95
	12 a.m	372	1,752	1,335	798	1,260	42	51	50	70	66	27	28	27	24	26	2.07	1.85	1.78	0.90	1.14
	2 p.m	291	1,341	866	1,050	1,160	39	48	56	49	66	28	29	26	27	26	2.31	2.08	1.48	1.82	1.14
	4 p.m	78	491	351	503	264	45	68	64	52	77	26	25	25	25	24	1.85	1.01	1.14	1.52	0.69

	**DFS**	**0**	**1**	**2**	**7**	**14**	**0**	**1**	**2**	**7**	**14**	**0**	**1**	**2**	**7**	**14**	**0**	**1**	**2**	**7**	**14**

Summer	8 a.m	385	806	994	793	908	85	67	69	89	71	24	29	30	29	30	0.45	1.32	1.32	0.44	1.23
	10 a.m	463	1,516	1,980	1,735	1,756	83	56	52	61	57	25	33	34	33	33	0.54	2.21	2.56	1.96	2.16
	12 a.m	574	1,661	1,226	2,000	269	79	50	50	50	65	26	34	34	36	30	0.71	2.66	2.66	2.97	1.49
	02 p.m	453	917	1,209	1,276	618	83	91	52	46	83	25	28	34	37	28	0.54	0.34	2.56	3.39	0.64
	04 p.m	187	366	477	763	190	72	71	66	54	75	26	29	31	33	28	0.94	1.16	1.53	2.32	0.95

*Days of exposure to full sunlight (DFS) 0, 1, 2, 7, and 22/14 were considered in two seasons, autumn/summer, respectively.*

On DFS 0, the PAR was extremely low, attaining the maximum values of 372 and 574 μmol m^–2^s^–1^ in autumn and summer, respectively, due to nursery shade conditions (Table 1). After exposure to full sunlight, in both seasons (autumn and summer), elevated PAR above 1,200 μmol m^–2^ s^–1^ occurred mainly between 12 a.m. and 2 p.m. The PAR recorded at 8 a.m. and 4 p.m. remained below 1,000 μmol m^–2^ s^–1^, in both seasons.

In autumn, at 8:00 a.m., RH always remained above 79%, decreasing over the diurnal cycle (Table 1). The lowest values of RH were registered at 12 a.m. and 2 p.m. In summer, RH remained above 46%, even in full sunlight. At 8 a.m., RH remained above 67%. On DFS 1 and 14 at 2 p.m., the presence of clouds contributed to the RH staying high, about 91 and 83%, respectively.

In autumn, the air temperatures remained between 17° and 30°C, whereas in summer they were higher, reaching 37°C (Table 1). In both the seasons, the hottest diurnal periods were at 12 a.m. and 2 p.m., with the highest temperatures registered on DFS 7 in summer, occurring together with the highest PAR registered (2,000 μmol m^–2^ s^–1^).

In autumn, VPD was between 0.90 and 2.31 kPa in the diurnal period of ecophysiological measurements, 12 a.m. to 2 p.m. (Table 1). In summer, the highest VPD was registered in the DFS 7 (3.39 kPa) in the same diurnal period.

### Thermography, Water Stress Index, and Index of Relative Stomatal Conductance

**FIGURE 1:**
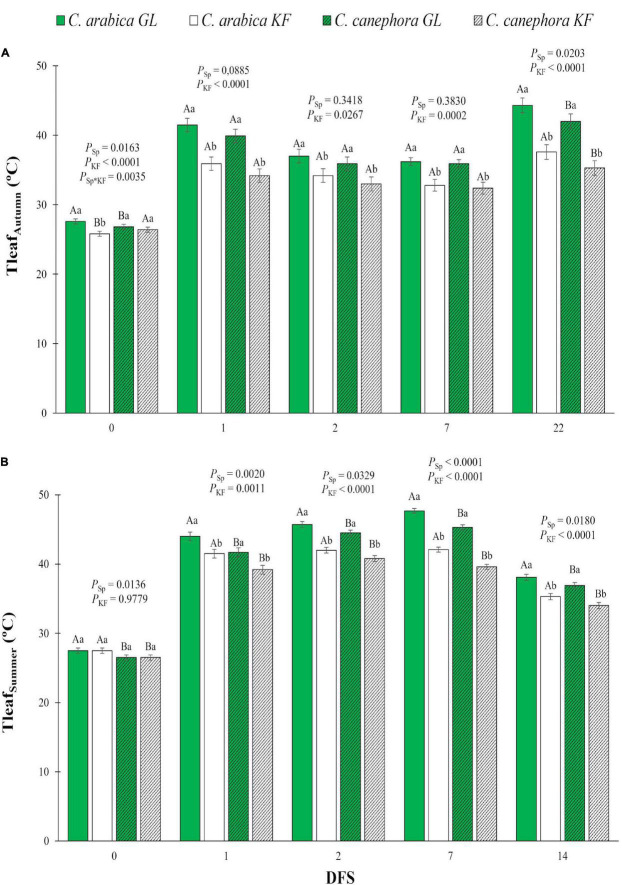
Leaf temperature (T_leaf_) estimated by thermography in young plants of two species (Sp), *Coffea arabica* and *C. canephora*, protected with kaolin film (KF) and not protected (GL), during the transference of coffee seedlings from nursery to full sunlight in **(A)** autumn (T_leaf Autumn_) and **(B)** summer (T_leaf Summer_). Means ± S.E. followed by different lowercase letters indicate statistically different values between kaolin treatments within the same species, while uppercase letters indicate differences between two coffee species within the same kaolin treatment, detected by the ANOVA and Tukey test (*n* = 5). *P*-values for effects of species kaolin and their interactions are indicated for days of exposure to full sunlight (DFS).

Thermography measurements were performed in totally expanded, most recently emitted leaves, in dates defined at 2.1 when all ecophysiological measurements were performed. They were effectuated at midday (12 a.m. to 2 p.m), which corresponded to the diurnal period of the highest PAR and the highest air temperature (Table 1). On experimental plants, one pair of recently matured leaves was used: one leaf was wetted with water on its adaxial face 5 min before the image was recorded, to reduce leaf temperature (Tl_eaf_) due to water evaporation from the leaf surface, representing T_wet_. On the second leaf, the Vaseline was applied (Costa et al., 2013) on its abaxial face (stomata are at abaxial side in coffee), 30 min before the image was registered, to attain the maximum T_leaf_, because of transpiration blockage, representing T_dry_ (Figure 1). The reference leaf temperature (*T*_*canopy*_) was measured on plants used for leaf gas exchange measurements. Leaves that received the Vaseline usually dropped after 5 days. For this reason, the thermography analyses were performed with additional 20 plants (*n* = 5) on each evaluation day, summing 100 plants for each season.

Thermal images were obtained with a Flir i50 mid-wave infrared camera (Flir Systems, Billerica, MA, United States) with camera emissivity set to 0.96. With a focal plane array detector, images with a resolution of 140 × 140 pixels (19,600 pixels, circles at Figure 1) were produced with an accuracy of ± 2%. For thermography measurements, the equipment was approached approximately 0.50 m above the plant. The captured images were stored in the equipment’s memory, and all image processing and analysis were undertaken in Flir Tools software version 5.2.15161 in the temperature range 20–50°C. Thermographic images were treated with the iron palette, using a circle, to calculate T_dry_, T_wet_, and T_canopy_ temperatures.

From the T_canopy_, T_dry,_ and T_wet_, the canopy CWSI was calculated. CWSI concept was developed by Idso et al. (1981), relating the observed temperature to the minimum (non-stressed) and maximum (non-transpiring) temperatures of a reference crop under similar environmental conditions. Its adaptation by Jones (2018), mitigates the downfalls of the original CWSI to the Equation 1:

(1) CWSI=(T-canopyT)wet/(T-dryT)wet

The thermal index of relative stomatal conductance (I_*g*_) was calculated on according to Jones (2018) using Eq 2:


(2) I=g(T-dryT)canopy/(T-canopyT)wet

### Leaf Gas Exchange and Chlorophyll *a* Fluorescence Measurements

The instantaneous leaf gas exchanges measurement included net CO_2_ assimilation rate (*A*, μmol CO_2_ m^–2^ s^–1^), transpiration (*E*, mmol H_2_O m^–2^ s^–1^), stomatal conductance (*g*_s_, mol m^–2^ s^–1^), instantaneous WUE (μmol mmol^–1^, calculated as the linear regression of *A/E*), and intrinsic water use efficiency (iWUE, μmol mol^–1^, calculated as linear regression of *A*/*g*_s_). They were performed on the same leaves as thermography, at midday (12 a.m.–2 p.m.), corresponding to the diurnal period of the highest PAR (Table 1) and the highest air temperature. The measurements were performed with an infrared gas analyzer LI-6400 (LI-COR, Lincoln, Nebraska, United States), with an external (CO_2_) supply of 400 μL L^–1^ and PAR of 1,500 μmol m^–2^ s^–1^ (from a 6400-02B, LED source composed on 80% red and 20% blue light), to attain leaf light saturation. The temperature and relative humidity inside the chamber were defined at 25°C and 60%, respectively.

In the KF treatment, immediately before the leaf-gas measurements, at the assessment site on the leaf blade, the kaolin particles were gently removed with cotton puffs to prevent their transition to the equipment pipes, avoiding undesirable modifications in the reading, and to assess the appropriate effect damage to the leaf mesophyll. Immediately after the readings, the leaves of the KF treatment received KF application, avoiding the exposure of the leaf tissue to the sunlight.

Chlorophyll *a* fluorescence measurements were performed in the same leaves and dates used for thermography and leaf gas exchange analyses at midday (12 a.m.–2 p.m.), using a non-modulated fluorimeter model Pocket PEA (Plant Efficiency Analyzer, Hansatech, King’s Lynn, Norfolk, United Kingdom). Leaves were previously dark-adapted for about 30 min, using Hansatech leaf clips. This premeasure ensures that all reaction centers of photosystem II (PSII) acquired an “open” status, and heat loss is minimalized (Strasser et al., 2000). Thereafter, the dark-adapted leaf parts were exposed to saturating irradiance of red light (650 nm, 3,500 μmol m^–2^ s^–1^, which is the technical limit of Pocket PEA) to obtain the fast chlorophyll *a* fluorescence transient of PSII, usually used to detect the stress impact affecting photosynthetic processes (Oukarroum et al., 2009). Subsequently, the collected data were submitted to the JIPtest (Strasser et al., 2004). Some variables generated by the JIPtest were used, such as the maximum PSII quantum yield (F_v_/F_m_), and the photosynthetic index (PI) (Strasser et al., 2004).

### Statistical Analyses

The analyses of the effects of KF (KF for kaolin film application or GL for green leaves), genotype (*C. arabica* and *C. canephora*), and their interactions in leaf gas exchange responses, chlorophyll *a* fluorescence, and thermography were performed *via* two-way analysis of variance (ANOVA) using R software (R Core Team, 2021). The “nlme” (Pinheiro et al., 2021), “emmeans” (Searle et al., 1980), and “agricolae” (De Mendiburu and Simon, 2015) packages were used. All data were previously evaluated for homogeneity of variance by the Bartlett’s test (Snedecor and Cochran, 1989). A linear mixed-effects model (LME) was used to perform ANOVA and the Tukey test for comparison of treatment means, at each of the two studied seasons (autumn or summer). Models were compared by the likelihood ratio test and, when appropriate, reduced models were adopted. The estimated means and standard errors (s.e.) are represented in tables and charts.

## Results

### Thermography Responses

After exposure to full sunlight, young plants protected with KF showed lower T_leaf_ than GL plants (Figure 1). KF caused a decrease in T_leaf_ up to 6.7°C for both species during autumn (Figure 1A). On DFS 22 in autumn, *C. arabica* showed T_leaf_ greater than *C. canephora* in both treatments. In summer, *C. canephora* plants managed to keep its leaves cooler than *C. arabica* (Figure 1B). KF impacted a T_leaf_ decrease of 5.6°C for *C. arabica* and 5.7°C for *C. canephora* compared with GL in summer.

### Leaf Gas Exchange Responses

*Coffea* sp. protected with KF reached higher *A* than GL on days 1, 2, and 7 DFS in both the seasons, whereas only in summer DFS 14 in *C. canephora* (Table 2). In summer, *A* was higher in *C. canephora* than in *C. arabica* on DFS 0, 2, 7, and 14. The average *A* increases in *C. arabica* protected with KF was 53 and 281% when compared with GL, whereas in *C. canephora* it was about 42 and 101% in autumn and summer, respectively. This means that *C. arabica* increased *A* more than *C. canephora* when protected with kaolin.

**TABLE 2:** The instantaneous leaf gas exchanges measurement included net CO_2_ assimilation rate (*A*, μmol CO_2_ m^–2^ s^–1^), stomatal conductance (*g*_s_, mmol H_2_O m^–2^ s^–1^), transpiration rate (*E*, mmol H_2_O m^–2^ s^–1^).

			*C. arabica*		*C. canephora*		*P*-values		
		DFS	GL	KF	GL	KF	Species	Kaolin	Sp. × KF
*A* (μmol CO_2_ m^–2^ s^–1^)	Autumn	0	6.74 ± 0.96 Aa	8.22 ± 0.96 Aa	8.57 ± 0.96 Aa	10.1 ± 0.96 Aa	0.1033	0.1784	–
		1	3.52 ± 0.70 Ab	6.59 ± 0.70 Aa	4.48 ± 0.66 Ab	7.55 ± 0.70 Aa	0.2533	0.0021	–
		2	5.04 ± 0.94 Ab	7.58 ± 0.95 Aa	5.12 ± 0.94 Ab	7.66 ± 0.94 Aa	0.9474	0.0345	–
		7	3.24 ± 0.80 Ab	5.23 ± 0.80 Aa	4.26 ± 0.80 Ab	6.25 ± 0.80 Aa	0.2686	0.0412	–
		22	3.59 ± 0.96 Aa	5.15 ± 0.96 Aa	5.51 ± 0.96 Aa	7.64 ± 0.96 Aa	0.0886	0.1583	–
	Summer	0	2.31 ± 1.18 Ba	3.17 ± 1.18 Ba	9.85 ± 1.18 Aa	10.7 ± 1.18 Aa	< 0.0001	0.4543	–
		1	1.04 ± 0.55 Ab	4.62 ± 0.55 Aa	2.17 ± 0.55Ab	5.74 ± 0.55 Aa	0.0752	< 0.0001	–
		2	0.40 ± 0.43 Bb	3.28 ± 0.43 Ba	1.71 ± 0.43 Ab	4.59 ± 0.43 Aa	0.0204	0.0001	–
		7	1.06 ± 0.45 Bb	3.72 ± 0.45 Ba	2.74 ± 0.45 Ab	5.40 ± 0.45 Aa	0.0032	0.0001	–
		14	2.42 ± 0.40 Ba	3.66 ± 0.40 Ba	4.58 ± 0.40 Ab	7.64 ± 0.40 Aa	0.0026	0.0501	0.0413
*g*_s_ (mol H_2_O m^–2^ s^–1^)	Autumn	0	0.073 ± 0.006 Bb	0.119 ± 0.017 Ba	0.118 ± 0.010 Ab	0.164 ± 0.018 Aa	0.0020	0.0186	–
		1	0.056 ± 0.014 Aa	0.062 ± 0.014 Aa	0.060 ± 0.007 Aa	0.066 ± 0.007 Aa	0.7357	0.4682	–
		2	0.009 ± 0.013 Bb	0.084 ± 0.005 Aa	0.049 ± 0.013 Aa	0.055 ± 0.005 Ba	0.0464	0.0001	0.0037
		7	0.029 ± 0.010 Bb	0.074 ± 0.010 Ba	0.054 ± 0.010 Ab	0.099 ± 0.010 Aa	0.0213	0.0004	–
		22	0.045 ± 0.019 Aa	0.079 ± 0.019 Aa	0.064 ± 0.019 Aa	0.098 ± 0.019 Aa	0.2538	0.0506	–
	Summer	0	0.001 ± 0.000 Bb	0.002 ± 0.000 Ba	0.012 ± 0.002 Ab	0.012 ± 0.002 Aa	< 0.0001	<0.0001	–
		1	0.056 ± 0.014 Aa	0.062 ± 0.014 Aa	0.061 ± 0.007 Aa	0.066 ± 0.007 Aa	0.7357	0.4682	–
		2	0.009 ± 0.013 Bb	0.084 ± 0.005 Aa	0.049 ± 0.013 Aa	0.054 ± 0.005 Ba	0.0464	0.0001	0.0037
		7	0.030 ± 0.014 Bb	0.103 ± 0.008 Aa	0.073 ± 0.014 Aa	0.086 ± 0.008 Aa	0.0391	0.0003	0.0126
		14	0.034 ± 0.009 Bb	0.074 ± 0.004 Ba	0.057 ± 0.009 Ab	0.097 ± 0.004 Aa	0.0015	0.0007	–
*E* (mmol H_2_O m^–2^ s^–1^)	Autumn	0	1.49 ± 0.11 Bb	2.05 ± 0.21 Ba	2.09 ± 0.15 Ab	2.65 ± 0.20 Aa	0.0017	0.0151	–
		1	1.35 ± 0.20 Aa	1.19 ± 0.20 Aa	1.10 ± 0.20 Aa	0.94 ± 0.20 Aa	0.4716	0.2658	–
		2	0.27 ± 0.33 Bb	1.94 ± 0.10 Ba	1.33 ± 0.33 Aa	1.37 ± 0.10 Aa	0.0426	0.0003	0.0048
		7	0.71 ± 0.18 Bb	1.55 ± 0.18 Ba	1.21 ± 0.18 Ab	2.05 ± 0.18 Aa	0.0063	0.0001	–
		22	1.16 ± 0.38 Aa	1.86 ± 0.38 Aa	1.61 ± 0.37 Aa	2.31 ± 0.37 Aa	0.1975	0.0532	–
	Summer	0	0.03 ± 0.02 Bb	0.10 ± 0.02 Ba	0.60 ± 0.09 Ab	0.67 ± 0.09 Aa	0.0001	0.0079	–
		1	0.51 ± 0.22 Bb	2.01 ± 0.22 Aa	1.87 ± 0.22 Aa	2.08 ± 0.22 Aa	0.0008	0.0004	0.0118
		2	0.84 ± 0.08 Bb	2.85 ± 0.24 Aa	2.56 ± 0.24 Aa	2.73 ± 0.26 Aa	< 0.0001	<0.0001	0.0013
		7	1.10 ± 0.46 Bb	3.07 ± 0.46 Aa	2.48 ± 0.46 Aa	2.78 ± 0.46 Aa	0.0064	0.0005	0.0156
			1.37 ± 0.34 Bb	2.70 ± 0.15 Ba	2.06 ± 0.34 Ab	3.39 ± 0.15 Aa	0.0052	0.0017	

*In young plants of two species (Sp.), Coffea arabica and C. canephora, protected with kaolin film (KF) and not protected (GL), during transference of coffee seedlings from the nursery to full sunlight in autumn and summer. Means ± S.E. followed by different lowercase letters indicate statistically different values between kaolin treatments within the same species, while uppercase letters indicate differences between two coffee species within the same kaolin treatment, detected by the ANOVA and Tukey test (n = 5). P-values for effects of species kaolin and their interactions are indicated for days of exposure to full sunlight (DFS).*

During autumn, KF increased *g*_s_ on DFS 0, 2, and 7 in *C. arabica*, whereas only on DFS 0 and 7 in *C. canephora* (Table 2). During summer, higher *g*_s_ in KF than in GL treatment was observed on DFS 0, 2, 7, and 14 in *C. arabica*, whilst only on DFS 0 and 14 in *C. canephora*. Furthermore, *C. canephora* plants had higher *g*_s_ than *C. arabica* on DFS 0 and 7 during autumn, and on DFS 0 and 14 during summer, regardless of the kaolin treatment. Interestingly, *C. canephora* leaves protected with KF had lower *g*_s_ than *C. arabica* on autumn and summer DFS 2, whereas the opposite situation was observed on the not protected leaves on the same DFS. With the KF protection, *C. arabica* increased the *g*_s_ for 229 and 264% compared with GL, whereas *C. canephora* for 39 and 24%, in autumn and summer, respectively. This means that the *g*_s_ increases in *C. arabica* were much higher than in *C. canephora*.

The impact of KF on *E* in *C. arabica* was greater in summer than in autumn (Table 2). In autumn, *C. arabica* plants protected with KF had higher *E* than GL treatment on DFS 0, 2, and 7, whereas in summer this positive effect on elevated *E* was observed during the whole observed period. *C. canephora* protected with KF maintained higher *E* than GL treatment on DFS 0 and 7 in autumn, and on DFS 0 and 14 in summer. On DFS 2 of autumn only in *C. arabica* increased *E* by KF, whereas this response was repeated on DFS 1, 2, and 7 of summer in a range of 179–620% (interaction Sp. × KF). *C. canephora* showed generally higher *E* values than *C. arabica*, regardless of the kaolin treatment, especially in summer. With the KF protection, *C. arabica* increased the *E* for 171 and 207% compared with GL, whilst *C. canephora* for 26 and 21%, in autumn and summer, respectively. This means that the *E* increases in *C. arabica* were up to tenfolds higher than in *C. canephora* in summer.

**FIGURE 2:**
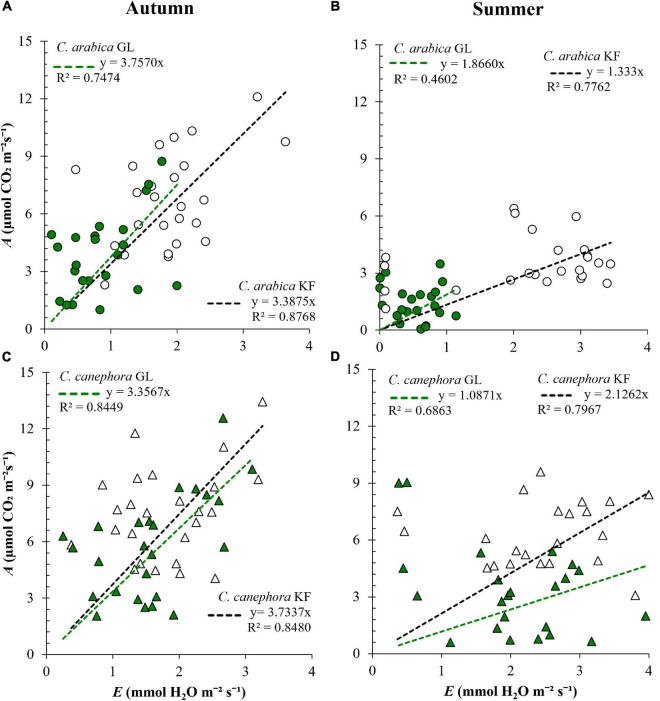
Instantaneous water use efficiency (WUE, μmol mmol^– 1^) in young plants of **(A,B**) *Coffea arabica* and **(C,D)** *C. canephora* protected with kaolin film (KF) and not protected (GL), during a transference of coffee seedlings from nursery to the full sunlight in autumn and summer.

Protection with KF increased *A*, *E*, and *g*_s_ in both species (Table 2). In *C. arabica* the increase in *E* was proportionally greater than the increase in *A*, resulting in WUE lower in KF plants than in GL plants by 9.83% in autumn and 28.56% in summer (Figures 2A,B). Similarly, the increase in *g*_s_ in *C. arabica* was proportionally greater than the increase in *A*, resulting in iWUE lower by 14.32% in autumn and 8.78% in summer compared with GL (Supplementary Figures 2A,B). *C. canephora* plants protected with KF maintained higher WUE than GL plants by 11.23% in autumn and 95.58% in summer (Figures 2C,D), and higher iWUE by 1.79% in autumn and 75.55% in summer, compared with GL plants (Supplementary Figures 2C,D). Higher iWUE and WUE responses with KF protection in *C. canephora* were the consequence of generally lower relative increases in *g*_s_ and *E* in this species when compared with *C. arabica*.

### Crop Water Stress Index and Index of Relative Stomatal Conductance

**FIGURE 3:**
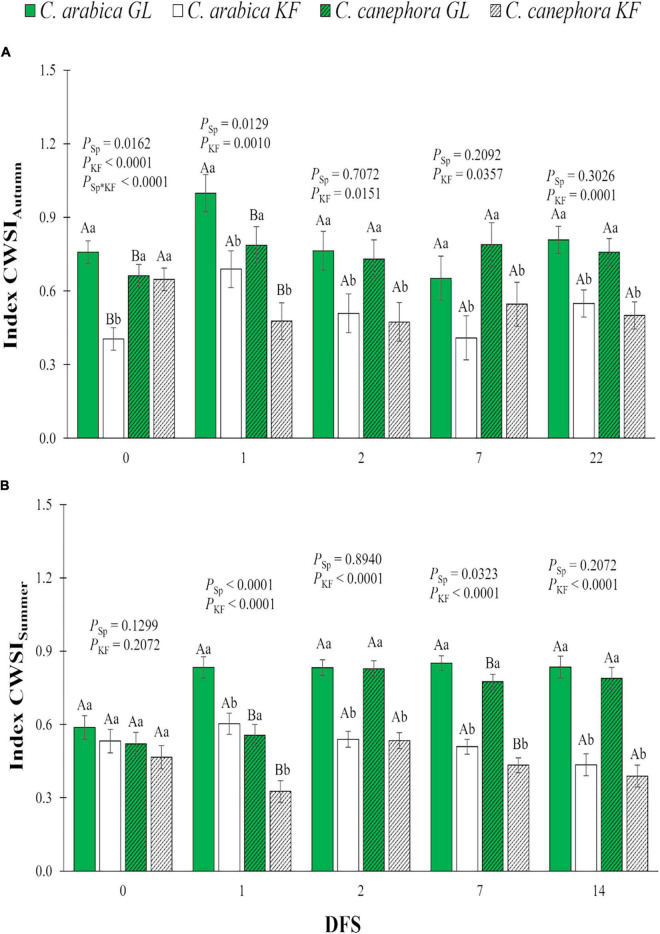
Water stress index (CWSI) in young plants of two species (Sp.), *Coffea arabica* and *C. canephora*, protected with kaolin film (KF) and not protected (GL), during transference of coffee seedlings from the nursery to the field in **(A)** autumn (CWSI_Autumn_) and **(B)** summer (CWSI_Summer_). Means ± S.E. followed by different lowercase letters indicate statistically different values between kaolin treatments within the same species, while uppercase letters indicate differences between two coffee species within the same kaolin treatment, detected by the ANOVA and Tukey test (*n* = 5). *P*-values for effects of species kaolin and their interactions are indicated for days of exposure to full sunlight (DFS).

The general response to KF was CWSI reduction in both *C. arabica* and *C. canephora* plants in autumn and summer on all DFS (Figure 3). In spite of that CWSI on DFS 0 in *C. arabica* estimated in autumn was lower in a group of plants destinated to KF application compared with the GF group, and the general tendency was CWSI increase in DFS 1 in all treatments (Figure 3A). *C. canephora* maintained lower CWSI than *C. arabica* on DFS 1 during autumn (Figure 3A) and on DFS 1 and 7 during summer (Figure 3B), regardless of the kaolin treatment.

**FIGURE 4:**
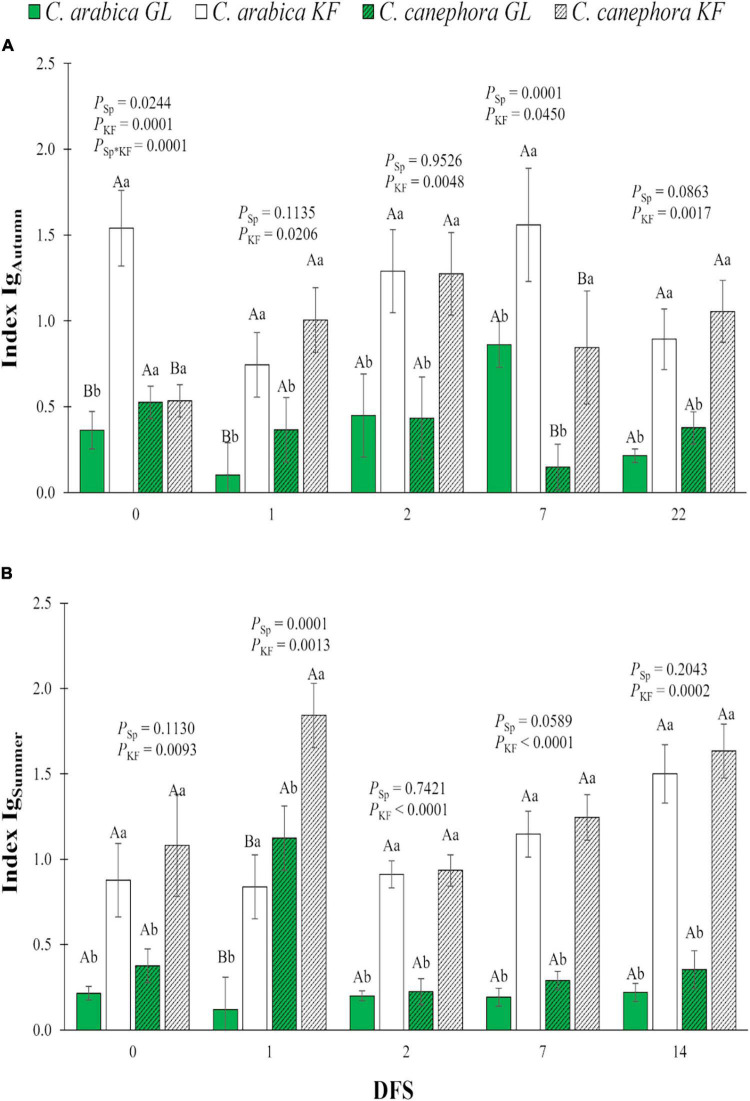
Index of relative stomatal conductance (I_g_) in young plants of two species (Sp.), *Coffea arabica* and *C. canephora*, protected with kaolin film (KF) and not protected (GL), during transference of coffee seedlings from the nursery to full sunlight in **(A)** autumn (I_gAutumn_) and **(B)** summer (I_gSummer_). Means ± S.E. followed by different lowercase letters indicate statistically different values between kaolin treatments within the same species, while uppercase letters indicate differences between two coffee species within the same kaolin treatment, detected by the ANOVA and Tukey test (*n* = 5). *P*-values for effects of species kaolin and their interactions are indicated for days of exposure to full sunlight (DFS).

The application of KF increased the I_g_ in both species on DFS 1, 2, 7, and 22 during the autumn (Figure 4A) and on DFS 0, 1, 2, 7, and 14 during the summer (Figure 4B). On DFS 7 in autumn, *C. arabica* had higher I_g_ than *C. canephora* (Figure 4A), whereas on DFS 1 in summer, *C. arabica* had lower I_g_ than *C. canephora* (Figure 4B), regardless of the kaolin treatment.

### Chlorophyll *a* Fluorescence

Protection with KF reduced the initial fluorescence (F_0_) when compared with GL on DFS 0, 1, 2, and 22 during autumn (Table 3), indicating better functioning of the PSII reaction centers in this treatment than in GL. In summer, this situation was observed on DFS 2 in both species and in *C arabica* on DFS 7. *C. arabica* had higher F_0_ than *C. canephora* on DFS 2 and 7 during autumn, and on DFS 14 during the summer, regardless of the kaolin treatment.

**TABLE 3:** The initial fluorescence (F_0_), maximum PSII quantum yield (F_v_/F_m_), and the photosynthetic index (PI) in young plants of two species (Sp), *Coffea arabica* and *C. canephora*, protected with kaolin film (KF) and not protected (GL), during transference of seedlings from the nursery to full sunlight, in autumn and summer.

			*C. arabica*		*C. canephora*		*P-values*		
		DFS	GL	KF	GL	KF	Species	Kaolin	Sp. × KF
*F*_0_	Autumn	0	5,791 ± 182 Aa	4,865 ± 295 Ab	5,372 ± 186 Aa	4,446 ± 304 Ab	0.1118	0.0096	–
		1	8,798 ± 650 Aa	5,886 ± 650 Ab	10,108 ± 650 Aa	7,196 ± 650 Ab	0.1044	0.0019	–
		2	6,077 ± 158 Aa	4,813 ± 158 Ab	5,073 ± 158 Ba	3,808 ± 158 Bb	< 0.0001	<0.0001	–
		7	6,409 ± 536 Aa	6,002 ± 253 Aa	5,563 ± 536 Ba	5,156 ± 253 Ba	0.0218	0.4573	–
		22	11,293 ± 795 Aa	4,753 ± 478 Ab	10,250 ± 640 Aa	3,710 ± 123 Ab	0.6843	0.0154	–
	Summer	0	4,712 ± 135 Aa	4,499 ± 97.8 Aa	4,582 ± 219 Aa	4,369 ± 198 Aa	0.5421	0.2067	–
		1	8,798 ± 650 Aa	5,886 ± 650 Ab	10,108 ± 650 Aa	7,196 ± 650 Ab	0.1044	0.0019	–
		2	5,555 ± 435 Aa	5,287 ± 714 Aa	4,657 ± 386 Aa	5,040 ± 172 Aa	0.1483	0.7541	–
		7	3,196 ± 409 Ba	5,964 ± 409 Ab	5,320 ± 409 Aa	4,303 ± 409 Ba	0.0015	0.0002	0.0002
		14	5,616 ± 186 Aa	5,386 ± 186 Aa	4,793 ± 186 Ba	4,563 ± 186 Ba	0.0020	0.3022	–
*Fv/Fm*	Autumn	0	0.77 ± 0.01 Ab	0.78 ± 0.01 Aa	0.77 ± 0.06 Ab	0.79 ± 0.01 Aa	0.4716	0.0410	–
		1	0.50 ± 0.04 Ab	0.63 ± 0.04 Aa	0.26 ± 0.04 Bb	0.40 ± 0.04 Ba	0.0002	0.0108	–
		2	0.55 ± 0.04 Aa	0.74 ± 0.04 Aa	0.49 ± 0.04 Aa	0.69 ± 0.04 Aa	0.2814	0.0009	–
		7	0.66 ± 0.03 Ab	0.67 ± 0.03 Aa	0.65 ± 0.03 Ab	0.67 ± 0.03 Aa	0.9502	0.4588	–
		22	0.71 ± 0.01 Ab	0.77 ± 0.01 Ba	0.30 ± 0.15 Bb	0.79 ± 0.01 Aa	0.0302	0.0048	0.0239
	Summer	0	0.78 ± 0.01 Aa	0.78 ± 0.01 Aa	0.79 ± 0.01 Aa	0.80 ± 0.01 Aa	0.2349	0.5115	–
		1	0.48 ± 0.04 Ab	0.67 ± 0.04 Aa	0.39 ± 0.04 Ab	0.57 ± 0.04 Aa	0.0733	0.0025	–
		2	0.55 ± 0.04 Ab	0.74 ± 0.04 Aa	0.49 ± 0.03 Ab	0.69 ± 0.04 Aa	0.2814	0.0009	–
		7	0.43 ± 0.05 Bb	0.59 ± 0.04 Ba	0.55 ± 0.03 Ab	0.71 ± 0.02 Aa	0.0118	0.0006	–
		14	0.60 ± 0.04 Aa	0.69 ± 0.02 Aa	0.63 ± 0.04 Aa	0.72 ± 0.02 Aa	0.2931	0.0642	–
*PI*	Autumn	0	4.64 ± 0.74 Ab	7.81 ± 1.02 Aa	4.97 ± 1.06 Ab	8.14 ± 1.31 Aa	0.7860	0.0176	–
		1	0.71 ± 0.26 Ab	1.26 ± 0.32 Aa	0.02 ± 0.01 Bb	0.57 ± 0.24 Ba	0.0196	0.0381	–
		2	0.61 ± 0.21 Ab	3.80 ± 0.60 Aa	0.56 ± 0.21 Ab	3.74 ± 0.60 Aa	0.8001	0.0001	–
		7	1.35 ± 0.33 Aa	1.39 ± 0.32 Aa	1.41 ± 0.33 Aa	1.46 ± 0.32 Aa	0.8078	0.8742	–
		22	2.71 ± 0.69 Ab	7.58 ± 0.94 Ba	0.51 ± 0.13 Bb	11.6 ± 1.14 Aa	0.0127	0.0025	0.0073
	Summer	0	4.26 ± 0.79 Aa	4.09 ± 0.79 Aa	4.57 ± 0.79 Aa	4.40 ± 0.79 Aa	0.7341	0.8542	–
		1	0.49 ± 0.19 Ab	1.51 ± 0.37 Aa	0.09 ± 0.04 Ab	1.11 ± 0.36 Aa	0.0511	0.0140	–
		2	0.44 ± 0.12 Aa	0.97 ± 0.32 Aa	1.00 ± 0.33 Aa	1.83 ± 0.50 Aa	0.1342	0.1431	–
		7	0.77 ± 0.21 Aa	1.67 ± 0.52 Aa	0.43 ± 0.21 Aa	1.32 ± 0.52 Aa	0.0688	0.0917	–
		14	0.43 ± 0.11 Ba	1.03 ± 0.27 Ba	1.07 ± 0.28 Aa	1.66 ± 0.34 Aa	0.0481	0.0562	–

*Means ± S.E. followed by different lowercase letters indicate statistically different values between kaolin treatments within the same species, while uppercase letters indicate differences between two coffee species within the same kaolin treatment, detected by the ANOVA and Tukey test (n = 5). P-values for effects of species, kaolin, and their interactions are indicated for days of exposure to full sunlight (DFS).*

Kaolin increased F_v_/F_m_ on DFS 0, 1, 2, and 22 in both species during autumn, whereas on DFS 1, 2, and 7 during summer (Table 3). On DFS 1 and 22 during autumn, the F_v_/F_m_ was higher in *C. canephora* compared with *C. arabica*, as on DFS 7 during the summer, regardless of the kaolin treatment.

Protection with KF increased PI in both species on DFS 0, 1, 2, and 22 during autumn and on DFS 1 during summer (Table 3). *C. arabica* plants had higher PI than *C. canephora* in the DFS 1 during the autumn regardless of the kaolin treatment, with no significant species impact in summer.

## Discussion

We first showed that during the transference of young *Coffea* plants from nursery shade to full sunlight, KF application decreased the stressed conditions of the new environment characterized by high light and elevated temperatures. Both species decreased T_leaf_, which impacted a general increase of leaf gas exchange parameters (*g*_s_, *E, A*) and I_g_. In both *Coffea* sp., KF application reduced F_0_, indicating reduced physical dissociation of the PSII reaction centers from the light-harvesting system (Sundby et al., 1986), which was supported with increased PI. Interestingly, only *C. canephora* leaves protected with KF achieved higher WUE compared with not-protected ones, which was one specific species response.

In cascade of plant responses during the transition from nursery to the sunlight, the KF application in young *Coffea* plants turned the leaf surfaces white (increasing PAR reflection), which firstly reduced T_leaf_ at the hottest daylight period up to 6.7°C during autumn, and up to 5.6°C during summer. Considering the effects of KF in other species, the reduction of T_leaf_ by 3°C is observed in grapefruit, *Citrus paradisi* (Jifon and Syvertsen, 2003), or by 2.5°C in rose, *Rosa* sp. (Sotelo-Cuitiva et al., 2011). The reduction in T_leaf_ is explained by the ability of KF to create a modified leaf/plant microclimate by the reflective nature of kaolin particles (Glenn et al., 2002; Steiman et al., 2007). The KF white color and formulation increase albedo on the fruit or leaf surfaces (Shellie and King, 2013), increasing radiation reflection on the canopy, impacting on T_leaf_ reduction (Campostrini et al., 2010), as was observed in both *Coffee* species, with high efficiency in midday.

The reduction in T_leaf_ of the KF-protected plants in *Coffea* sp. occurred in parallel with the increase in *g*_s_, *E*, and I_g_, and a reduction in CWSI. The microclimate created by the application of KF reduced the possible negative environmental effects of high PAR and high T_air_, minimizing the partial or total closure of the coffee stomata, as happened without KF technology application (Martins et al., 2014; DaMatta et al., 2019). Without KF, *C. canephora* kept the leaves cooler at midday than *C. arabica*, which was likely related primarily to the higher *E* values linked to higher *g*_s_, knowing that the increased transpiration rate results in increased latent heat loss and reduced leaf temperature (Ainsworth and Rogers, 2007; Jones, 2018). Additionally, overall, non-KF treated leaves from *C. canephora* have showed higher stomatal density than *C. arabica* (Rodrigues et al., 2018; Bernado et al., 2021), helping to understand the species-specific responses, i.e., could allow *C. canephora* leaves to respond more rapidly to changing environmental cues. *C. arabica* also showed increased *g*_s_ and *E* values when treated with KF, but the difference in *g*_s_ and *E* between the two coffee species was reduced with KF spraying, resulting from the reduction in *C. canephora* efforts to acclimatize on high light and temperature.

The *A* was fluctuated in two seasons, in well-watered *Coffee* plantlets, with generally higher assimilation in autumn than in summer as previously observed in Rodrigues et al. (2016). The season in adult *Coffee* plants grown in field conditions without irrigation can produce the opposite effect, showing higher assimilation in rainy summer than in dry autumn (Rakocevic et al., 2021b). Generally, *A* was higher in *C. canephora* than in *C. arabica* in plants protected with KF than in not protected, which was associated with increases in *g*_s_. In fact, kaolin reduces abscisic acid accumulation in grapevine leaves, helping in the faster recovery of leaf gas exchanges under high light and temperature (Dinis et al., 2018), which was probably the mechanism of biochemical action in young coffee plants.

Photosynthetic carbon assimilation increased in *C. canephora* and *C. arabica* when protected with KF, but in *C. arabica* the increases in *E* and *g*_s_ were proportionally greater than the increases in *A*, and therefore, the WUE and iWUE were reduced compared with GL plants. In summer, *C. canephora* protected with KF increased *A* more than *E*, resulting in elevated WUE when compared with GL plants, or to *C. arabica* protected with KF. A similar response is observed in grapevine, where KF application reduces canopy temperature and the thermal stress, impacting on increased WUE and productivity (Glenn et al., 2010). On the other hand, *C. arabica* decreased WUE and iWUE in both seasons due to relatively higher increases in *g*_s_ and *E* than in *A*, when compared with *C. canephora*. Kaolin applied at high doses acts as an antitranspirant, impacting the direction of leaf *A* and *E* reductions in some stages of grapevines (Frioni et al., 2019, 2020). Two coffee species differ in anatomical leaf characteristics: *C. arabica* is characterized by a greater thickness of the abaxial epidermis and the spongy parenchyma, and by the lower thickness of the palisade parenchyma and reduced stomatal density than *C. canephora* (Bernado et al., 2021). Considering those anatomical species specificities and their differential responses in leaf gas exchanges with KF application, the question is: Could kaolin spraying dose be different between *C. arabica* and *C. canephora* species to provoke a positive response in water savings? In future research, reduced doses could be tested in *C. arabica* to promote WUE elevation, and water savings. The elevated WUE in *C. canephora* in the summer period can lead to water savings. In fact, the KF application in other species, such as in strawberry (*Fragaria ananassa*) seedlings during transplanting, allowed savings between 20 and 40% of the water volume without affecting plant growth and green intensity (Santos et al., 2012). The protective effect of KF places this technology as a sustainable development tool to mitigate the effects of ongoing global warming and allows water economy (Roy et al., 2018).

When leaves are submitted to heat stress, the increase of chlorophyll fluorescence (F_0_ parameter) is observed (Smillie and Nott, 1979). KF influenced the chlorophyll fluorescence emission and minimized damage to the photochemical apparatus before the appearance of visual symptoms in these two coffee species, *C. canephora* and *C arabica* (data not shown). *Coffea* sp. plants protected with KF had lower F_0_, higher F_v_/F_m_, and higher PI than those not protected. This effect presumably reflects the physical dissociation of the PS II reaction centers from the light-harvesting system (Sundby et al., 1986). F_v_/F_m_ values less than 0.75 indicate a photo-inhibitory effect of the PSII-associated photosynthetic apparatus (Bolhar-Nordenkampf et al., 1989), which occurred in coffee exposed to full sunlight, regardless of KF treatment. The PI values of coffee seedlings not protected with KF indicated that the activity of PS I and PS II was compromised during the transition of the seedlings from a nursery shade to full sunlight. In *C. canephora* protected with KF, PI values were upto 25-fold higher than those not protected with KF on DFS 2 during the summer. Results about coffee seedlings not protected with KF may suggest some destabilization of membranes and proteins, production of reactive oxygen species, and cell death, as observed in apples (Gindaba and Wand, 2007).

In conclusion, the application of KF on coffee leaves would reduce T_leaf_ under high PAR and high T_air_ during the sensitive agronomic management of young *Coffea* plants, confirming the initial hypothesis. KF impacted on F_0_, F_v_/F_m_, and PI modifications in *Coffea* sp., minimizing possible damages of the photochemical apparatus, preventing the stomatal closure, and permitting higher net CO_2_ assimilation. For the *C. arabica*, it seems that autumn can be considered as the best season for planting, although KF application improved the plant acclimatization to elevated light and temperatures at midday. On the other hand, *C. canephora* showed greater plasticity than *C. arabica* related to the planting season. Observing the species-specific responses in water management efficiency with KF applications, *C. canephora* showed higher WUE and iWUE than *C. arabica*, indicating water savings in *C. canephora* cultivations, from the practical point of view. The second practical point of view could be related to diminished costs and risks, where the dilution of 1 kg of KF (the commercial price of Surround WP is about 4–5 USD) in 20 L of water (5% w/v) can protect 1,550 m^2^ of nursery bed or around 300,000 to 450,000 young coffee plantlets. In the field, 1 kg of KF in 5% w/v protects 1 hectare, i.e., 3,000–5,000, of newly planted coffee plants. Thus, the processed-kaolin particle film technology is important in the transition of seedlings from the nursery to the field planting condition, given that the young plant price is 0.12–0.20 USD, whereas the KF cost per plant is less than 0.002 USD. The use of KF can be used as a management strategy to protect leaves from the two coffee species against excess solar radiation, elevated temperatures, and excess water spend, especially in summer and in *C. canephora*.

## Data Availability Statement

The raw data supporting the conclusions of this article will be made available by the authors, without undue reservation.

## Author Contributions

DA: investigation, data curation, and writing original draft. NR: resources and conceptualization. GA: software and data curation. WB: investigation. WR: methodology. EC: resources, conceptualization, definition, and validation. MR: conceptualization, validation, reviewing, and editing. All authors read and approved the final manuscript.

## Conflict of Interest

The authors declare that the research was conducted in the absence of any commercial or financial relationships that could be construed as a potential conflict of interest.

## Publisher’s Note

All claims expressed in this article are solely those of the authors and do not necessarily represent those of their affiliated organizations, or those of the publisher, the editors and the reviewers. Any product that may be evaluated in this article, or claim that may be made by its manufacturer, is not guaranteed or endorsed by the publisher.

## Abbreviations

T_air_: Air temperature
VPD: air vapor pressure deficit
CWSI: canopy water stress index
DFS: days of exposure to full sunlight
KF: fine particle film based on calcined and purified kaolin
F_0_: initial fluorescence
WUE: instantaneous water use efficiency
iWUE: intrinsic water use efficiency
Tl_eaf_: leaf temperature
F_v_/F_m_: maximum photochemical efficiency
*A*: net CO_2_ assimilation rate
PI: photosynthetic index
PS I and PS II: photosystems I and II
PAR: photosynthetically active radiation
RH: relative humidity
*g*_s_: stomatal conductance
I_g_: thermal index of relative stomatal conductance
*E*: transpiration.
