# Supplementary material for: Kaolin Film Increases Gas Exchange Parameters of Coffee Seedlings During Transference From Nursery to Full Sunlight
Source: Front Plant Sci. 2022 Jan 7;12:784482. doi: 10.3389/fpls.2021.784482 (PMC8777232; doi:10.3389/fpls.2021.784482)
Supplement: Supplementary file 1 [file Data_Sheet_1.docx]

Supplementary Material

**Kaolin in Coffee Seedlings Acclimatization During Transference from Nursery to Full Sunlight**
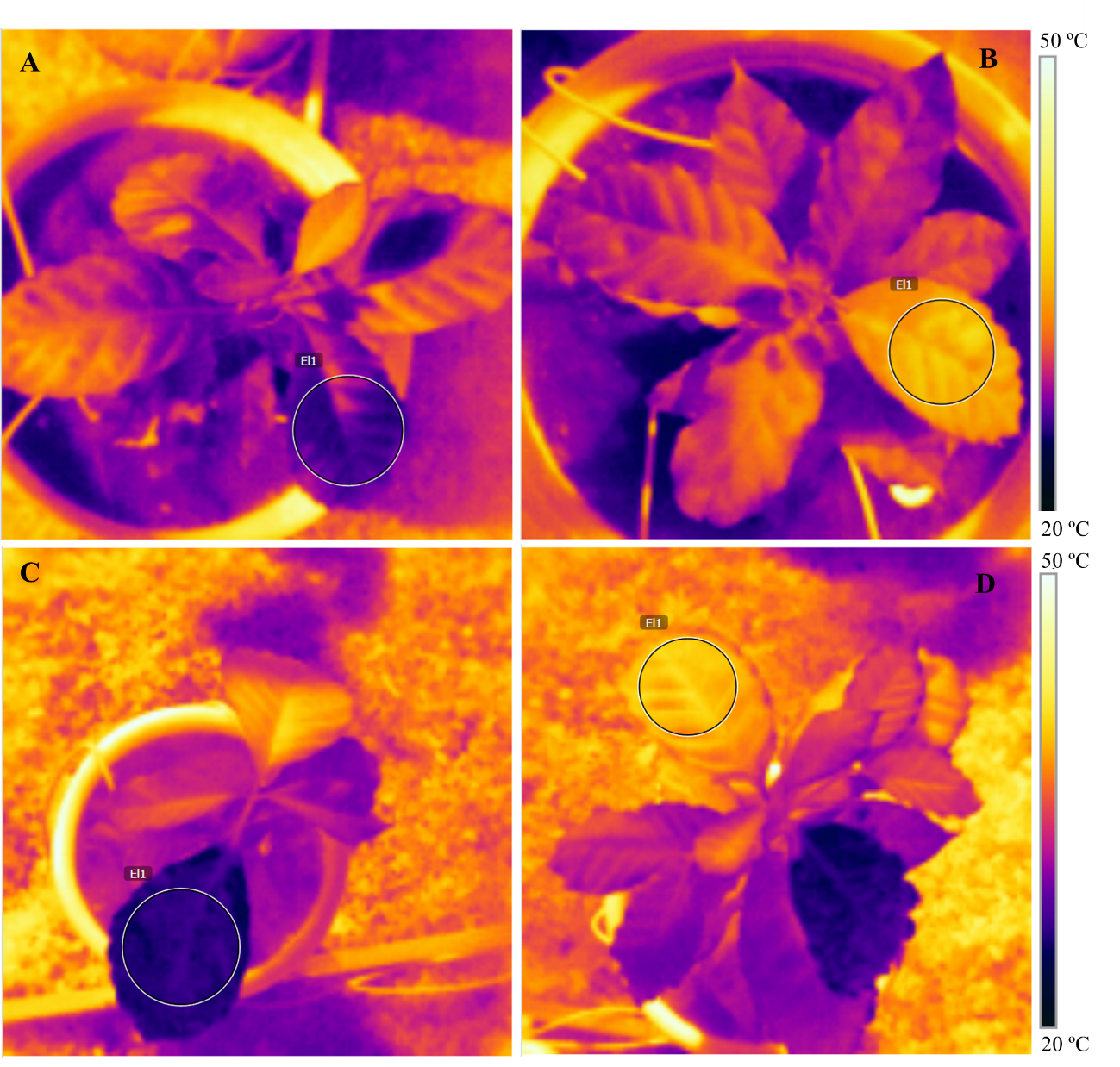


**Supplementary Figure S1**. Thermal images obtained with an infrared camera in **A**) and **B**) *Coffea arabica* and **C**) and **D**) *C. canephora*. The leaves were moistened: **A**) and **C**) with water on abaxial surface, representing T_wet_, **B**) and **D**) with Vaseline on the adaxial surface, representing T_dry_.


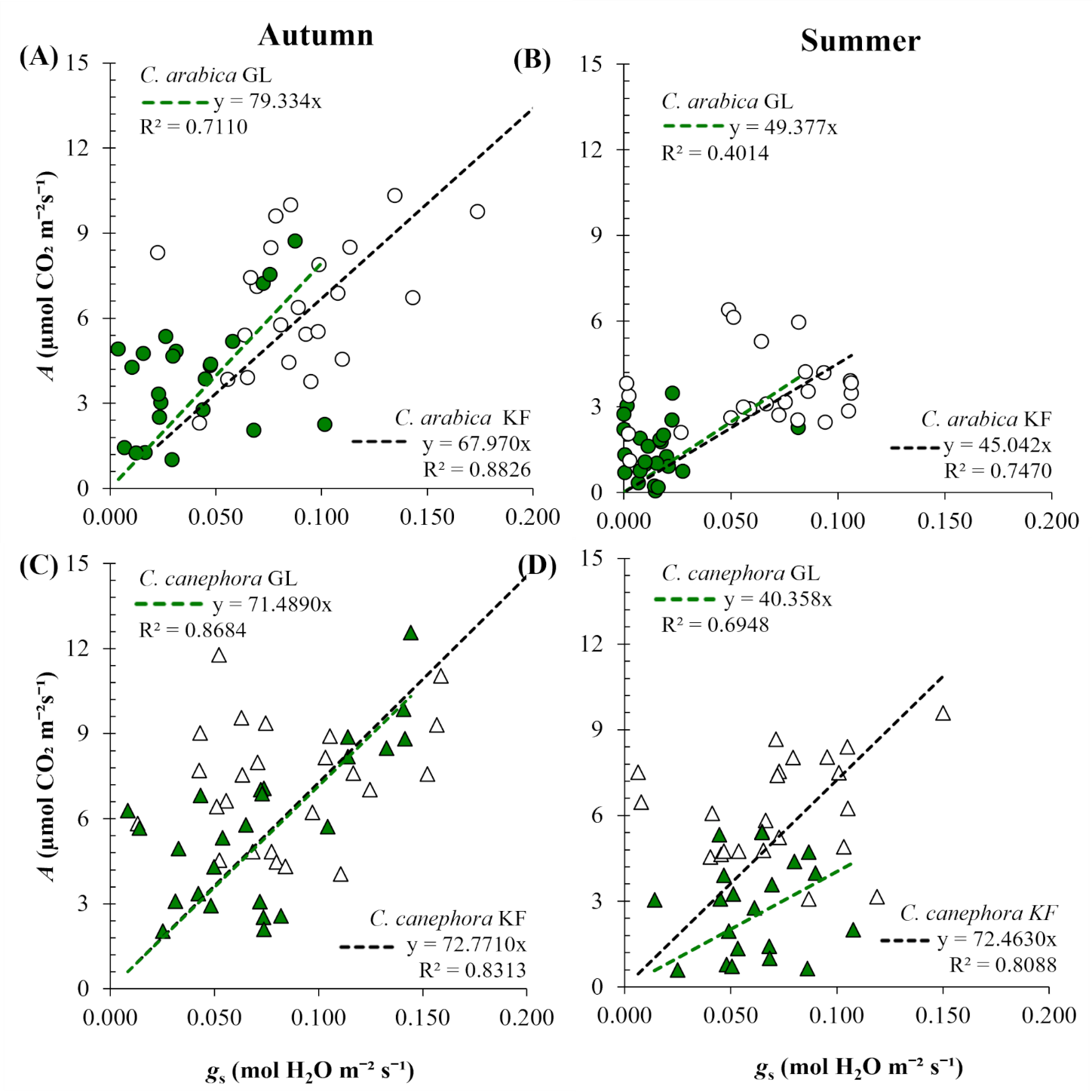


**Supplementary Figure S2**. Leaf water use efficiency (iWUE, µmol mol^–1^), in young plants of *Coffea arabica* (**A** and **B**) and *C. canephora* (**C** and **D**), protected with kaolin film (KF) and not protected (GL), during a transference of coffee seedlings from nursery to the field in autumn and summer.
